# Threats of Bots and Other Bad Actors to Data Quality Following Research Participant Recruitment Through Social Media: Cross-Sectional Questionnaire

**DOI:** 10.2196/23021

**Published:** 2020-10-07

**Authors:** Rachel Pozzar, Marilyn J Hammer, Meghan Underhill-Blazey, Alexi A Wright, James A Tulsky, Fangxin Hong, Daniel A Gundersen, Donna L Berry

**Affiliations:** 1 Phyllis F Cantor Center for Research in Nursing and Patient Care Services Dana-Farber Cancer Institute Boston, MA United States; 2 School of Nursing University of Rochester Rochester, NY United States; 3 McGraw/Patterson Center for Population Sciences Dana-Farber Cancer Institute Boston, MA United States; 4 Department of Psychosocial Oncology and Palliative Care Dana-Farber Cancer Institute Boston, MA United States; 5 Department of Data Sciences Dana-Farber Cancer Institute Boston, MA United States; 6 Survey and Data Management Core Dana-Farber Cancer Institute Boston, MA United States; 7 Department of Biobehavioral Nursing and Health Informatics University of Washington Seattle, WA United States

**Keywords:** social media, internet, methods, data accuracy, fraud

## Abstract

**Background:**

Recruitment of health research participants through social media is becoming more common. In the United States, 80% of adults use at least one social media platform. Social media platforms may allow researchers to reach potential participants efficiently. However, online research methods may be associated with unique threats to sample validity and data integrity. Limited research has described issues of data quality and authenticity associated with the recruitment of health research participants through social media, and sources of low-quality and fraudulent data in this context are poorly understood.

**Objective:**

The goal of the research was to describe and explain threats to sample validity and data integrity following recruitment of health research participants through social media and summarize recommended strategies to mitigate these threats. Our experience designing and implementing a research study using social media recruitment and online data collection serves as a case study.

**Methods:**

Using published strategies to preserve data integrity, we recruited participants to complete an online survey through the social media platforms Twitter and Facebook. Participants were to receive $15 upon survey completion. Prior to manually issuing remuneration, we reviewed completed surveys for indicators of fraudulent or low-quality data. Indicators attributable to respondent error were labeled suspicious, while those suggesting misrepresentation were labeled fraudulent. We planned to remove cases with 1 fraudulent indicator or at least 3 suspicious indicators.

**Results:**

Within 7 hours of survey activation, we received 271 completed surveys. We classified 94.5% (256/271) of cases as fraudulent and 5.5% (15/271) as suspicious. In total, 86.7% (235/271) provided inconsistent responses to verifiable items and 16.2% (44/271) exhibited evidence of bot automation. Of the fraudulent cases, 53.9% (138/256) provided a duplicate or unusual response to one or more open-ended items and 52.0% (133/256) exhibited evidence of inattention.

**Conclusions:**

Research findings from several disciplines suggest studies in which research participants are recruited through social media are susceptible to data quality issues. Opportunistic individuals who use virtual private servers to fraudulently complete research surveys for profit may contribute to low-quality data. Strategies to preserve data integrity following research participant recruitment through social media are limited. Development and testing of novel strategies to prevent and detect fraud is a research priority.

## Introduction

Health research participants are increasingly recruited online [1]. Researchers may access potential research participants through a variety of online sources, including classified advertisements, search engine advertisements, survey panels, email listservs, crowdsourced online labor markets, and social media platforms [2-4]. Recruitment of health research participants through social media is particularly popular and has been reported in at least 69 unique papers published between 2011 and 2019 [5-7].

Recruitment of health research participants through social media may appeal to researchers for several reasons. First, 80% of US adults use social media, and rates of social media use exceed 60% in almost every sociodemographic category for which data are available [8]. Although only 40% of US adults aged 65 years and older use social media, this proportion has grown substantially from 12% in 2010 [8]. Second, social media platforms permit researchers to target advertisements to users according to their age, gender, education, location, interests, and behaviors [9]. Targeted social media advertisements enable researchers to direct their recruitment efforts toward individuals who are likely to meet study eligibility criteria. Third, the practical and ethical considerations of recruiting health research participants from social media have been well characterized. Guides to using social media to recruit participants to health research studies are available in the peer-reviewed literature and are increasingly produced by academic institutions [9-14]. Likewise, several authors have proposed approaches to ensure the protection of human research participants who are recruited through social media [11,13,15].

Researchers have sought to describe the extent to which participant recruitment through social media is cost-effective and efficient [2-4,7,16-19]. Although study results vary, some researchers suggest the use of social media may be more efficient and affordable than traditional recruitment methods in clinical settings [5]. Likewise, there is evidence that social media platforms effectively provide researchers with a way to access members of small or difficult-to-reach populations [7,11,12,16,20]. Despite these findings, studies in which research participants are recruited through social media are vulnerable to the same challenges associated with other methods of recruiting research participants online [21,22]. Respondent misrepresentation of eligibility criteria, duplicate enrollment, and automated enrollment by software applications known as bots pose serious threats to sample validity and data integrity [23]. Nevertheless, these challenges are poorly described in the health sciences literature, particularly as they relate to the recruitment of health research participants through social media.

Ongoing development of best practices for all aspects of online research is necessary to encourage rigor and ensure judicious use of limited resources. The purpose of this paper is to describe and explain potential threats to sample validity and data integrity associated with the recruitment of health research participants through social media. We use our recent experience recruiting health research participants through social media as a case study. Drawing upon this example and from published research within and outside of the health sciences literature, we aim to provide a comprehensive overview of strategies that may be used to mitigate these threats.

## Methods

We designed a cross-sectional descriptive study that elicited patient perceptions of patient-provider communication in the ovarian cancer care setting. At the time of initial recruitment, eligible participants were English-speaking US adults diagnosed with ovarian cancer within the last 12 months. We planned to recruit participants through the Facebook and Twitter social media platforms and collect data online through a Research Electronic Data Capture (REDCap) survey [24]. Upon survey completion, valid participants would be issued a $15 electronic gift card. The Dana-Farber/Harvard Cancer Center institutional review board (IRB) approved the study protocol.

**Figure 1:**
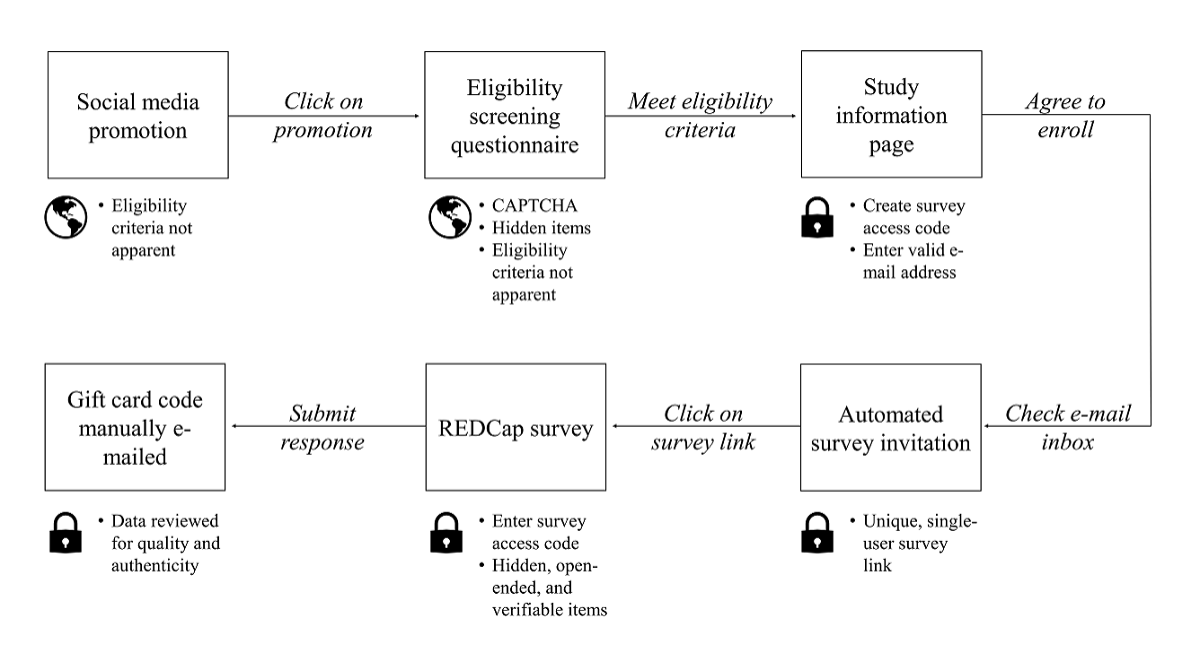
Procedure for participant recruitment and enrollment.

We developed our study protocol (Figure 1) after reviewing published guides to recruiting health research participants online [9,11,23,25] and seeking advice from our institution’s REDCap administrators and survey research core. First, we created a study page on Facebook and study account on Twitter. Next, we developed and planned to disseminate a set of Facebook posts, targeted Facebook advertisements, tweets (Twitter posts), and targeted Twitter advertisements. The Facebook page, Twitter account profile, Facebook posts, tweets, and targeted advertisements each included a brief overview of the study purpose and link to an eligibility screening questionnaire. Promotions described a gynecologic cancer communication study rather than an ovarian cancer communication study to prevent respondent misrepresentation of eligibility criteria [9,11]. No other details related to eligibility criteria were apparent from study promotions.

To access the eligibility screening questionnaire, respondents were required to pass a completely automated public Turing test to tell computers and humans apart (CAPTCHA) [23,25]. The eligibility screening questionnaire asked respondents to report how they heard about the study and used branching logic to deny access to ineligible respondents [25]. Respondents who reportedly met eligibility criteria were directed to a study information page that included all elements of informed consent. The study information page informed respondents that remuneration was limited to one gift card per participant and evidence of fraudulent activity may result in study removal [23]. Respondents who agreed to enroll in the study were prompted to provide their email address and create a survey passcode. Enrolled participants received automated emails containing a unique survey link and were required to enter their passcode to access the survey.

The survey included 124 closed- and 14 open-ended items. We pretested the survey and estimated that it would require 15 minutes to complete. We designed the survey to include several elements aimed at identifying low-quality or fraudulent responses. These included (1) a timestamp at the beginning and end of the survey, (2) hidden items, which are visible to bots but invisible to human respondents, and (3) pairs of items that could be used to identify inconsistent or illogical responses (eg, timestamp time zone and self-reported location). Prior to manually distributing participant remuneration, we planned to review completed surveys for evidence of inattention, duplicate or unusual responses to open-ended items, inconsistent responses to verifiable items, and evidence of automation. Specific examples from each of these categories are provided in the Results section.

We initiated recruitment with a single tweet that read “Help researchers learn about communication in gynecologic cancer care. Fill out a research survey from Dana-Farber Cancer Institute and receive a $15 Amazon gift card. Visit [link to the eligibility screening questionnaire] to learn more.” We also added the link to the eligibility screening questionnaire to the study Facebook page and Twitter account profile. We scheduled targeted advertisements to be launched at a later date.

## Results

Less than 7 hours after initiating recruitment, 576 respondents had completed the eligibility screening questionnaire. We suspected fraudulent activity after noting that although eligibility was limited to US residents, 82.5% (475/576) of responses to the eligibility screening questionnaire were submitted between the hours of midnight and 4:00 am Eastern Standard Time. In turn, we removed the tweet containing the link to the eligibility screening questionnaire, deleted the link from the Facebook page and Twitter account profile, and temporarily deactivated the survey.

Of the respondents who completed the eligibility screening questionnaire, 47.0% (271/576) reportedly met eligibility criteria, enrolled in the study, and completed the survey. Of the completed surveys, 47.2% (128/271) were submitted between the hours of 1:00 and 5:00 am in the participant’s reported time zone. The mean time to survey completion was 12.8 (SD 14.8) minutes. Three members of the study team (RP, MJH, and DLB) assessed completed surveys for quality and authenticity. We began by highlighting evidence of inattention, duplicate or unusual responses to open-ended items, inconsistent responses to verifiable items, and evidence of automation in each case. Next, we documented the specific indicators of low-quality or fraudulent data that were present in the data set. Indicators that could reasonably be attributed to respondent error or coincidence were labeled as suspicious, while those that strongly suggested automation or respondent misrepresentation were labeled as fraudulent (Table 1). Given the possibility that some legitimate respondents could have completed the survey between the hours of 1:00 and 5:00 am, we opted not to include hour of survey submission on our list of indicators.

We classified cases with 1 fraudulent indicator or at least 3 suspicious indicators as fraudulent, cases with no fraudulent indicators and 1 to 2 suspicious indicators as suspicious, and cases with no fraudulent or suspicious indicators as legitimate.

In total, we classified 94.5% (256/271) as fraudulent, 5.5% (15/271) as suspicious, and none as legitimate. Most cases (241/271, 88.9%) exhibited more than 1 type of indicator of low-quality or fraudulent data. Of the fraudulent cases, 52.0% (133/256) exhibited evidence of inattention, with survey completion times under 5 minutes in 24.6% (63/256) of cases and under 10 minutes in 27.3% (70/256) of cases. More than half of the fraudulent cases (138/256, 53.9%) included a duplicate or unusual response to an open-ended item. For example, in response to an item asking if participants wished to share anything else about communicating with doctors and other health professionals, 2 respondents entered “professional and technical personnel carry out film packaging management.” In response to an item asking participants what recommendations their clinicians had made about surgery, 6 respondents entered “the first choice surgery excision treatment, surgery pathology.”

**Table 1:** Indicators of low-quality or fraudulent data.

Indicator		Designation
**Evidence of inattention**		
	Survey completion time <5 minutes	Fraudulent
	Survey completion time <10 minutes	Suspicious
	Same response provided to every closed-ended item on a survey page (straight lining)	Suspicious
**Duplicate or unusual responses to open-ended items**		
	Exact response (consisting of more than 2-3 words) provided by more than one respondent	Fraudulent
	Response is nonsensical or irrelevant to item	Suspicious
	Several responses follow the same pattern in terms of phrasing or formatting	Suspicious
	Response is an exact duplicate of text found on an existing website	Suspicious
**Inconsistent responses to verifiable items**		
	Reported location and zip code prefix do not match	Suspicious
	Reported location and timestamp time zone do not match	Suspicious
	Reported treatment facility is not a cancer care facility	Suspicious
	Timestamp time zone indicates survey was completed outside of the United States	Fraudulent
	Response to “Where did you hear about this survey?” identified an organization that was not involved with recruitment	Suspicious
**Evidence of bot automation**		
	Response provided to one or more hidden items	Fraudulent

In total, 86.7% (235/271) of cases included an inconsistent response to 1 or more verifiable items, and 16.2% (44/271) included a response to a hidden item. Every case that included a response to a hidden item and had valid timestamp data (25/271, 9.2%) exhibited a survey completion time under 2 minutes.

After consulting with our institution’s IRB, we removed fraudulent cases from the study without remuneration. We issued remuneration to the 15 respondents whose cases were classified as suspicious; however, we will exclude these cases from planned data analyses. We reinitiated recruitment by creating a duplicate REDCap project with a new URL. The new URL was not posted publicly; rather, promotions were limited to targeted Facebook advertisements and Facebook posts in private groups. Several months after successfully reinitiating recruitment in this fashion, we received 3 completed surveys in rapid succession. Upon review, we classified these cases as fraudulent. On review of Facebook user engagement with our targeted advertisements, we determined that a Facebook user who met our targeting criteria had shared one of our advertisements in a public Facebook post. We promptly removed the advertisements from Facebook and reinitiated recruitment using a third REDCap project URL without further issues.

## Discussion

### Principal Findings

Our initial attempt to recruit health research participants through social media resulted in a large volume of low-quality and fraudulent data. Although we implemented strategies to prevent respondent misrepresentation of eligibility criteria and automated enrollment, hundreds of respondents navigated past checkpoints meant to restrict access to eligible human respondents.

Although our study protocol was informed by published guidance on the recruitment of health research participants through social media [9-14], discussions of data quality and authenticity are largely absent from these works. Likewise, existing discussions of data quality and authenticity may be embedded in articles that discuss the challenges of online research more generally [23,25]. Researchers who consult the literature prior to recruiting health research participants through social media may overlook articles that do not refer to social media explicitly.

Our experience suggests studies in which research participants are recruited through social media are susceptible to many of the same pitfalls as studies in which participants are recruited through other online means [25-28]. In a related example, Dewitt and colleagues [22] conducted a cross-sectional descriptive study in which data were collected via web-based survey. The study team recruited research participants through an electronic mailing list and Facebook. Following data collection, they found that 60.5% (289/478) of completed survey responses were fraudulent. Similarly, Ballard and colleagues recruited research participants through an unspecified social media platform [21]. Following data collection, they determined that of the survey responses, 28.3% (117/414) were fraudulent and 10.1% (42/414) were potentially fraudulent. It is possible that the proportion of fraudulent responses was higher in our study because we shared the link to our eligibility screening questionnaire on both Facebook and Twitter. Nevertheless, these findings highlight the need to address issues of sample validity and data integrity as they pertain to the role of social media in health research.

Although issues of data quality and authenticity are not unique to studies in which research participants are recruited online, individuals who intend to defraud researchers may find that technology permits them to do so on a larger scale than would otherwise be possible. For example, bots can be programmed to rapidly complete online surveys. However, our experience and those of others suggest that the majority of fraudulent data cannot be attributed to bots alone [21,28]. All respondents in our study were able to pass a CAPTCHA, and only 16.2% (44/271) responded to one or more hidden survey items. Although some bots may be capable of passing a CAPTCHA and generating a fraudulent email address [22], access to our survey was restricted to respondents who provided a valid email address and had access to its inbox. Moreover, most respondents successfully identified a cancer treatment facility in the United States and entered a zip code prefix in the same geographic region. These activities require a degree of sophistication characteristic of human respondents [28].

Several authors have observed that *satisficing*, in which eligible respondents expend the minimal amount of cognitive effort needed to complete a survey, contributes to low-quality data [29,30]. One limitation of our fraud detection protocol is the overlap between indicators of fraud and indicators of satisficing. However, given the speed with which we accumulated low-quality data, it is likely that our results largely reflect a coordinated effort by ineligible respondents to obtain remuneration rather than sample-wide satisficing. Groups of individuals who intend to defraud researchers may exchange information about online research studies that provide financial incentives [23]. Moreover, in a 2019 blog post, the founder of a company specializing in market research identified at least one website dedicated to training individuals to fraudulently complete large volumes of online surveys [31]. Although the phenomenon of respondent misrepresentation has been identified in the health sciences literature [25], the mechanisms by which low-quality and fraudulent survey responses are submitted by human respondents are poorly described. Improved understanding of this phenomenon is necessary to prevent the exploitation of research studies in which participants are recruited through social media and other online means.

### Role of Virtual Private Servers

Research from other disciplines offers insight into strategies used to defraud researchers who recruit research participants online. In the field of behavioral accounting, Dennis and colleagues [28] described 2 studies in which they recruited research participants through Amazon’s Mechanical Turk, a crowdsourced online labor market. They received a large volume of responses that exhibited many of the same indicators of low-quality or fraudulent data that were present in our data. The authors used internet protocol (IP) address geolocation to identify the source of these responses and discovered multiple IP addresses with identical global positioning system coordinates. On further investigation, the authors determined that the IP addresses in question were associated with both a server farm and an internet service provider known to provide virtual private servers (VPSs).

**Figure 2:**
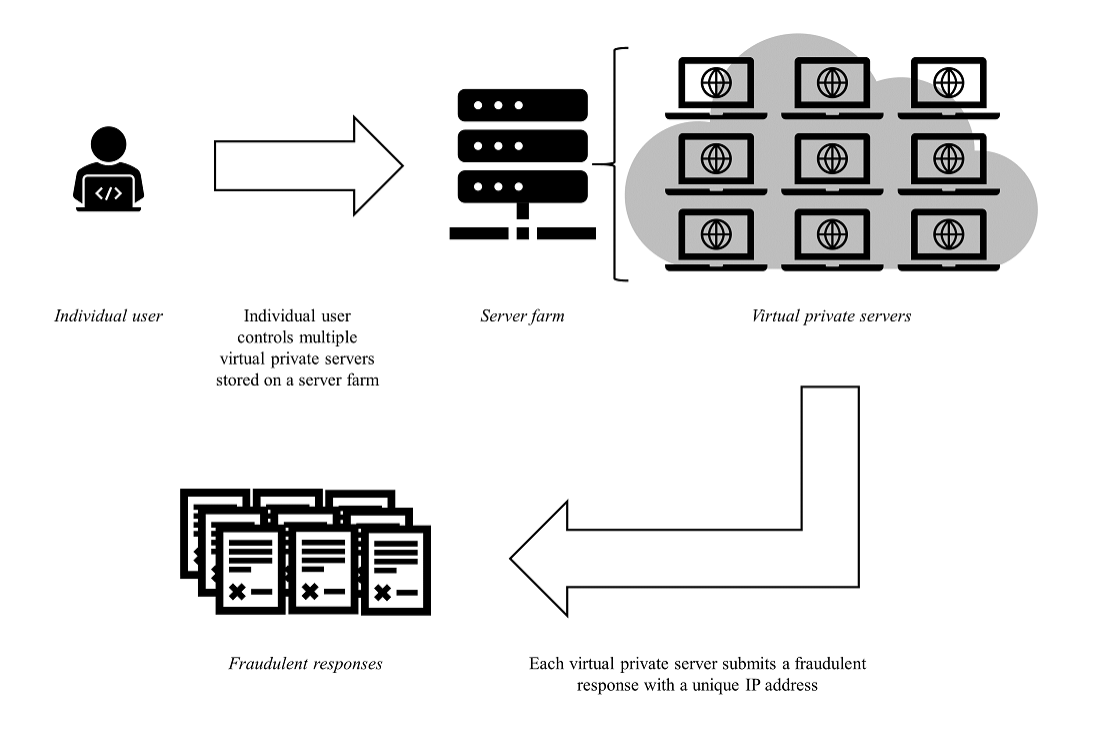
The role of virtual private servers in research participant misrepresentation.

Server farms are large collections of computer hardware housed in a single location. Server farms provide users with remote access to hardware with a processing capacity that exceeds that of a single computer. Each server farm can host a nearly unlimited number of VPSs, each of which functions like an individual computer but lacks its own physical hardware [28]. Like a physical computer, a VPS comprises data files, software programs, and an operating system [28]. An individual using more than one VPS would be able to use one physical computer to remotely program multiple VPSs to complete research surveys at the same time (Figure 2).

Responses that originate from one individual using more than one VPS may be difficult to identify. Each VPS has a unique IP address associated with the physical location of the server farm rather than that of its user [28]. An individual using more than one VPS may masquerade as multiple respondents, each with a unique IP address. Furthermore, an individual using a VPS hosted on a server farm within the United States may mask his or her true location and circumvent strategies to limit study enrollment to US residents [28,32].

A VPS is not the only way in which an individual can mask his or her location. Virtual private networks, anonymous proxies, and spoofed IP addresses may be used for the same purpose. It is important to note that some individuals conceal their location or IP address out of privacy concerns and may not have malicious intent [32]. However, research suggests VPS use is associated with the collection of low-quality and fraudulent data following online recruitment of research participants.

Dennis and colleagues [28] used respondent IP addresses to compare the data they received from respondents who used a VPS to the data they received from those who did not. In open-ended item responses, respondents who used a VPS exhibited significantly higher proportions of English language misuse, incoherent or nonsensical phrases, duplicate responses, and responses that were copied verbatim from an existing website. The similarities between the responses received by Dennis and colleagues and our study team suggest individuals who use a VPS to defraud researchers are active outside of crowdsourced labor markets and may enroll in research studies that recruit participants through social media. The implications of this finding for data quality are especially concerning given a recent analysis by Kennedy and colleagues [32], who analyzed 38 studies that recruited research participants through Amazon’s Mechanical Turk and found evidence of respondents using a VPS as early as 2015.

### Strategies to Preserve Sample Validity and Data Integrity

Published papers within and outside of the health sciences literature offer suggestions to avoid collecting low-quality and fraudulent data from research participants recruited online. Although most strategies are applicable to studies that recruit research participants through social media, we provide additional suggestions that are specific to this approach. Limited research describes strategies to identify respondents using a VPS. Herein, we summarize the progress that has been made in this area to date and identify topics in need of further development. Strategies to prevent collection of low-quality or fraudulent data are proposed according to project phase below.

Preparation of study protocol and IRB application:

- Develop a written protocol for identifying and responding to low-quality data [22]
- Include language that permits the study team to verify respondent identities if needed (eg, via telephone call) [22,25]
- In consent document, state that participants will be removed from the study without remuneration in cases of fraud and participants will not receive additional remuneration for completing the study more than once [21,23]
- Mail remuneration to a physical address to avoid respondent misrepresentation of location-based eligibility criteria [21,25]
- Lower the value of or eliminate remuneration [23,25]
- Prepare study advertisements that do not explicitly state eligibility criteria [9,11]
- Seek guidance from institutional resources (eg, information systems, research computing, and the IRB)

Preparation of data collection instruments:

- Use a data collection platform with fraud prevention and detection features (eg, Qualtrics) [21-23,32]
- Use automated invitations to send each respondent a unique link to the data collection instrument [25]
- Ask respondents to identify where they heard about the study [25]
- Require respondents to pass a CAPTCHA [22,23,25]
- Collect respondent IP addresses (according to the Health Insurance Portability and Accountability Act Privacy Rule, IP address is considered an identifier) [21,23,25,28,32,33]
- Collect verifiable information, such as telephone number or physical address [21,23]
- Include at least one hidden item in each instrument. This can be accomplished by adding the @HIDDEN action tag to an item in REDCap or by adding custom JavaScript code to an item in Qualtrics
- Include a time stamp at the beginning and end of each instrument [21,23,25]
- Include (and consider requiring a response to) open-ended items [28]
- Include items with embedded directives (eg, “select the third option below”) [27]
- Include pairs of items that can be compared for consistency [23,25]
- Include items that require respondents to demonstrate insider knowledge [25]

Active recruitment and data collection:

- Avoid posting links to data collection instruments in the public space
- Use targeted advertisements to avoid promoting the study to ineligible respondents [9]
- Limit visibility of study-related social media profiles to audiences in the target geographic regions
- Monitor social media user engagement with study posts and advertisements (eg, for public shares or comments related to eligibility criteria)
- Monitor frequency and content of responses for suspicious patterns
- Identify respondents using a VPS with a tool such as the rIP R package or Shiny [32-34]

Researchers will need to weigh the potential benefits of each strategy against the financial and practical burden it may impose. For example, eliminating participant remuneration may remove the incentive for individuals who aim to defraud researchers [23,25]. However, survey completion and response rates are likely to be higher when remuneration is offered [35]. Entering participants into a raffle drawing for a larger incentive may serve as an acceptable compromise [25]. Alternatively, to verify that respondents meet location-based eligibility criteria, researchers may elect to mail gift cards to a physical address rather than send them electronically [25]. Some researchers have reported successfully verifying respondent eligibility over the telephone [22,25], but as Teitcher and colleagues [23] observed, respondent eligibility verification is labor-intensive and may increase burden for legitimate participants.

Not every strategy mentioned will be appropriate for every research study. Similarly, no strategy will effectively preserve sample validity and data integrity when used alone. For example, although CAPTCHAs are intended to differentiate human respondents from bots, they are not always effective [22,23]. Likewise, although IP addresses can be used to verify that a respondent meets geographic eligibility criteria, IP-based geolocation is not always accurate [21,25]. Given that each strategy may be associated with one or more shortcomings, we recommend a comprehensive and multifaceted approach.

There is a need for research that develops and tests strategies to limit enrollment of individuals who may be using a VPS to defraud researchers. One approach has been proposed by Waggoner and colleagues [33], who developed a package called rIP for the statistical computing environment R (R Foundation for Statistical Computing). The rIP package provides researchers with the location of respondent IP addresses, information about likely VPS or server farm use, and a recommendation about whether to include the respondent’s data in the data set. The team created an online version of the tool called Shiny that allows users to upload comma-separated value files for analysis in lieu of using R [33]. Although the rIP package and Shiny application have the potential to substantially reduce the workload associated with data quality review, prevention of low-quality responses is preferable to retrospective data classification. In a separate paper, Kennedy and colleagues [32] described embedding code in their Qualtrics survey to identify respondents whose IP address is associated with a server farm or VPS. The code used the IP verification website IP Hub [36] to identify these respondents and redirected them to a message informing them that they were ineligible to participate in the study. Additional solutions that capitalize on emerging knowledge of low-quality and fraudulent data sources are needed.

### Limitations

Our study team did not collect the IP addresses of respondents. As such, we could not use the rIP R package or Shiny app [33] to determine whether a respondent used a VPS to access our survey. Future research that compares information provided by the rIP R package or Shiny app to the indicators of fraudulent or low-quality data that are described in this paper is warranted.

### Conclusions

The recruitment of health research participants through social media is associated with several potential advantages. Nevertheless, studies in which research participants are recruited through social media are vulnerable to significant threats to sample validity and data integrity. There is a pressing need for best practices to prevent respondent misrepresentation of eligibility criteria and to identify low-quality and fraudulent data. As health researchers increasingly turn to social media to access potential research participants, development of strategies to ensure rigor remains a priority.

## Abbreviations

CAPTCHA: completely automated, public Turing test to tell computers and humans apart
IP: internet protocol
IRB: institutional review board
REDCap: Research Electronic Data Capture
VPS: virtual private server
